# Heat, health and hatchlings: associations of *in situ* nest temperatures with morphological and physiological characteristics of loggerhead sea turtle hatchlings from Florida

**DOI:** 10.1093/conphys/coaa046

**Published:** 2020-06-03

**Authors:** Kelsey A Fleming, Justin R Perrault, Nicole I Stacy, Christina M Coppenrath, Alison M Gainsbury

**Affiliations:** 1 Department of Biological Sciences, University of South Florida St. Petersburg, St. Petersburg, FL 33701, USA; 2 Loggerhead Marinelife Center, Juno Beach, FL 33408, USA; 3 Aquatic, Amphibian, and Reptile Pathology Program, Department of Comparative, Diagnostic, and Population Medicine, College of Veterinary Medicine, University of Florida, Gainesville, FL 32608, USA

**Keywords:** Climate change, hematology, incubation temperatures, performance, reference interval, self-righting response

## Abstract

Incubation temperatures, in addition to an embryo’s genetic makeup, are critical in many aspects of adequate sea turtle embryonic development. The effects of high and low incubation temperatures on hatchling quality have been previously examined; however, many of these studies were conducted on relocated or laboratory-reared nests, which do not accurately reflect natural nest temperature fluctuations. To observe the impacts of varying *in situ* incubation temperatures on loggerhead sea turtle (*Caretta caretta*) hatchling morphology, various health variables and locomotor performance, temperature data loggers were deployed in 15 loggerhead nests on Juno Beach, Florida, between May and July 2018. Over the course of the study period, 10 morphological traits were measured, blood analytes and heart rate were assessed for the establishment of reference intervals and the self-righting response in seawater was evaluated. Warmer months were associated with smaller body size and higher body condition index, larger umbilical scar size, slower righting time, lower heart rates and higher packed cell volume, hemoglobin, total solids, total white blood cell count, absolute heterophils and absolute basophils. These findings provide evidence that higher *in situ* incubation temperatures have the potential to adversely affect hatchlings from warmer nests due to increased risk of predation from smaller body sizes, decreased physical responses and overall fitness, altered hemodynamic balance (e.g. dehydration) and potential inflammation and/or stress. With rising temperatures, we predict sea turtle hatchlings may have increasing risks of developing suboptimal physiological features affecting overall fitness and ultimately survival. These results demonstrate that rising environmental temperatures can negatively impact sea turtle hatchlings, thus representing additional stress on sea turtle populations and contributing to our understanding of potential pathophysiological effects of climate change on the delicate life-stage class of the sea turtle hatchling. This information will be useful for formulating effective future sea turtle management plans.

## Introduction

Apart from genetics, morphological and physiological traits of developing reptile embryos are notably influenced by incubation temperatures. Increases in incubation temperatures reportedly impact embryonic metabolism (Ligon and Lovern, 2012), immune function (Dang *et al*., 2015), reproductive success, hatchling body condition and locomotor performance (Elphick and Shine, 1998; Du and Ji, 2003; Booth *et al*., 2004; Tang *et al*., 2012). Additional effects of increased incubation temperatures include alterations in growth rates (Rhen and Lang 1995), yolk conversion rates (Booth, 2000; Booth and Astill, 2001; Booth *et al*., 2004) and behavior (Vervust *et al*., 2011; Siviter *et al*., 2017). By 2015, Earth’s atmospheric temperatures had risen 1°C above pre-industrial levels and are expected to increase anywhere from 0.3°C to 4.7°C, with an increase of at least 0.5°C by 2052 at the current rate (IPCC, 2018). The effects of ‘high’ and ‘low’ incubation temperatures on sea turtle hatchling size, quality and performance have been examined previously in other studies; however, many of these investigations were conducted on laboratory-reared nests or relocated nests. Laboratory-reared nests are often exposed to constant incubation temperatures not reflecting diurnal variations and other naturally varying environmental conditions such as humidity throughout the incubation period. Additionally, relocated nests frequently contain only a fraction of the eggs from the entire clutch, resulting in differences in metabolic heating and water uptake (Booth *et al*., 2004; Fisher *et al*., 2014).

Hatchling size is one of the most common traits known to be affected by incubation temperatures. Elevated incubation temperatures result in reduced mass, carapace lengths and fore and hind limb areas in leatherbacks (*Dermochelys coriacea*), loggerheads (*Caretta caretta*) and green turtles (*Chelonia mydas*) (Glen *et al*., 2003; Mickelson and Downie, 2010; Read *et al*. 2012; Sim *et al*. 2015). Incubation temperatures can also influence yolk conversion rates. As the yolk sac becomes internalized and nutrients are transferred from the yolk to the developing embryo, the embryo increases in size while the yolk decreases, resulting in the closing of the umbilicus (Keller, 2017). Eggs laid in cooler nests incubate longer and convert more yolk material to tissue, thus resulting in hatchlings with a larger yolk-free mass (Booth, 2000; Booth and Astill, 2001; Hewavisenthi and Parmenter, 2001; Glen *et al*., 2003; Booth and Evans, 2011).

Similar to hatchling size, the influence of nest incubation temperatures on hatchling performance is well documented. Optimal locomotor performance post-hatching is critical as it can impact the hatchlings’ ability to evade beach and near-shore predators and to survive in the open ocean. This ability to survive is directly related to their locomotor performance and, given the probability of predation is directly proportional to the time spent in coastal waters (Gyuris, 1994), the ability of a hatchling to quickly upright itself is vital. To date, most researchers have evaluated self-righting response on land (Booth *et al*., 2012; Read *et al*. 2012; Fisher *et al*., 2014; Wood *et al*., 2014; Sim *et al*., 2015); however, to the authors’ knowledge, righting response time in water has not been observed. The implications of the righting response in sea water are equally relevant to sea turtle hatchlings, as they can be overturned in the near-shore waves. Loggerhead hatchlings produced from relocated nests in Australia performed best in locomotor performance tests (e.g. faster crawl times and swim speeds) when the mean 3-day maximum incubation temperature was below 34°C (Sim *et al*., 2015). Additionally, Fisher *et al*. (2014) found that when loggerhead sea turtle eggs from South Carolina, USA were incubated at constant temperatures, ranging from 27–33°C, the optimal incubation range for peak performance on righting, crawling and swimming tests was 28.5–31.0°C. A similar optimal swimming performance range (28–30°C) was reported for olive ridley hatchlings (*Lepidochelys olivacea*) from Mexico when incubated at constant temperatures ranging from 24–34°C (Mueller *et al*., 2019). Given that incubation temperatures are frequently higher than 31°C in southeast Florida, with annual loggerhead maximum nest temperatures ranging from 34.2–36.5°C (Hanson *et al*., 1998; Wyneken and Lolavar, 2015), it is possible hatchlings from nests laid during peak nesting season (June–July, when environmental temperatures are typically high) in Florida will have sub-optimal locomotor performance.

To our knowledge, no published research exists regarding baseline blood data (e.g. hematology, biochemistry) in sea turtle hatchlings at time of emergence from the nest. These blood analytes can indicate various underlying conditions such as inflammation, dehydration, stress, disease and/or anemia and serve as important diagnostic indicators in sea turtles (Stacy and Innis, 2017). Certain blood analytes presumptively correlate with water temperature in sea turtles (Stamper *et al*., 2005; Campbell, 2006; Kelly *et al*., 2015; Perrault *et al*., 2016), but how this relates to incubation temperature is unknown. Incubation temperatures can also impact heart rates of developing reptiles, which in turn might influence oxygen delivery to tissues and locomotor performance (Booth, 2017). Embryonic heart rates reportedly increase with ambient and body temperatures in various oviparous reptiles including lizards, snakes, crocodilians and freshwater turtles (Gatten, 1974; Du *et al*., 2010, 2011). The influence of incubation temperatures on sea turtle hatchling cardiovascular development that could potentially result in heart rate differences post-emergence has yet to be evaluated (Booth, 2017).

**Figure 1:**
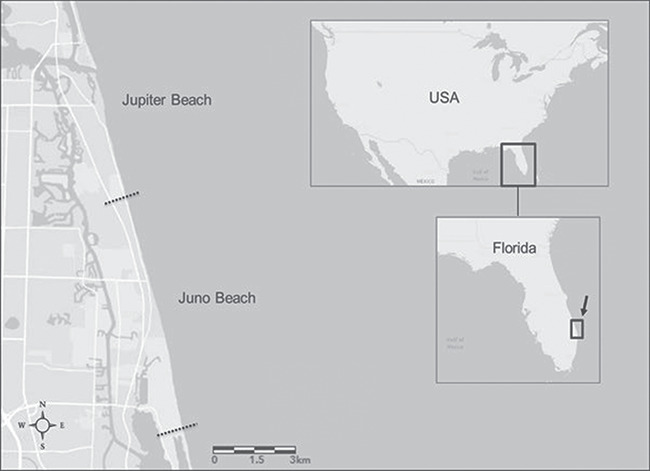
The study site for this project was Florida’s east coast on Juno Beach located in northern Palm Beach County, Florida, USA. The north and south end of the study site are represented by dotted lines

Our study objective was to describe the impacts of varying *in situ* incubation temperatures on loggerhead sea turtle hatchling morphology, various health variables and locomotor performance. We first investigated the influence of incubation temperatures on hatchling morphology including mass, minimum straight carapace length (SCL_min_), straight carapace width (SCW), body depth (BD), front flipper length (FFL) and umbilical scar size. Next, we analyzed hatchling blood analytes [e.g. whole blood glucose, packed cell volume (PCV), hemoglobin (Hbg), total solids, white blood cell (WBC) concentrations] and heart rates in correlation with incubation temperatures. Finally, we observed the effect of incubation temperatures on hatchling locomotor performance, specifically the self-righting response time in sea water.

## Materials and methods

This study was carried out with the approval of the University of South Florida’s Institutional Animal Care and Use Committee (#WIS00004563). Research activities were conducted under Florida Fish and Wildlife Conservation Commission (FFWCC) Marine Turtle Permit #205.

### Study site and collection

The sampling site was located along Florida’s east coast (Juno Beach, Florida, USA), primarily in the 9.63 km area of peak loggerhead sea turtle nesting activity (northernmost and southernmost nests ranged from 26.903492119°N, −80.059868605°W to 26.838829657°N, −80.042031936°W, respectively; Fig. 1). Approximately 90% of the nesting effort of this loggerhead subpopulation (northwest Atlantic) occurs on the peninsular region of Florida, making it the largest nesting population in the western hemisphere (Ehrhart *et al*., 2003, 2014; Ceriani *et al*., 2012, 2017). Additionally, Palm Beach County, Florida (where Juno Beach is located) experienced the highest number of loggerhead nests in all Florida counties from 2013–19 (FFWCC, 2019, FFWCC, 2020).

Nesting season of loggerhead sea turtles in Florida occurs from April through September and peaks from June to July. Onset® Hobo® Water Temperature Pro v2 data loggers (Onset Computer Corporation Bourne, Massachusetts, USA) were placed in the center of 15 clutches when nesting females were halfway through oviposition in May (*n* = 5 nests), June (*n* = 5 nests) and July (*n* = 5 nests) during the 2018 nesting season. These data loggers have an operational range of −40°C to 70°C in air with an accuracy of ±0.21°C from 0°C to 50°C. The data loggers were placed on 8 and 9 May, 13 and 14 June and 11 and 12 July into the middle of each clutch after ~50–60 eggs had been laid (an average of 116 ± 6 eggs were laid in the clutches included in this study) and were programmed to record temperature to 0.01°C every 30 min for the duration of incubation. Three days after the mass hatchling emergence event, nests were excavated and data loggers were removed.

Overall mean incubation temperature (T_inc_) was calculated for each nest, along with the mean temperature in the middle third of incubation (T_mid_), the mean temperature during the last 2 weeks of incubation (T_2w_) and the mean 3-day maximum temperature (T_3dm_). All encountered nesting females were tagged using a combination of passive integrated transponder (PIT; Biomark®, Inc., Boise, Idaho, USA) and Inconel flipper tags (National Band and Tag Co., Newport, Kentucky, USA) to ensure each clutch came from a different female. Nests were monitored for hatchling emergence starting at 40–45 d of incubation; hatchlings were collected as they naturally emerged from their nests. Up to 10 hatchlings from each clutch were collected during the mass emergence event (*n* = 144 hatchlings total; May = 50 hatchlings, June = 44 hatchlings, July = 50 hatchlings). Upon collection, hatchlings were placed in coolers with damp sand for transportation to Loggerhead Marinelife Center’s (LMC) Research Laboratory for testing. All sampling was immediately conducted at a constant ambient temperature and with all sources of artificial white light eliminated. Red light emitting diode headlamps were used during the sampling process.

### Morphological measurements

Mass was recorded to the nearest 0.01 g using an electronic scale. Measurements were collected using stainless steel digital calipers (±0.01 mm) and included SCL_min_, SCW, BD (all after Wyneken, 2001), FFL on the right front flipper from the radial condyle to the tip of the flipper at the third phalange (Wyneken, 2001) and umbilical scar length and width. Using the umbilical scar as a proxy for yolk mass, with a smaller umbilical scar serving as a proxy for a smaller yolk mass (Perrault, unpublished data), we investigated the effects of average incubation temperature on yolk size without having to sacrifice hatchlings. A carapace size index (CSI) was calculated by multiplying SCL_min_ by SCW (Sim *et al*., 2015). Body condition index (BCI) was also calculated using the following formula:

$$ \mathrm{BCI}=\left(\frac{\mathrm{mass}}{{{\mathrm{SCL}}^3}_{\mathrm{min}}}\right)\times 10\ 000\ \left(\mathrm{after}\ \mathrm{Bjorndal}\ \mathrm{et}\ \mathrm{al}.,2000\right) $$

### Health variables

Heart rate was taken manually using a portable ultrasound (EI Medical Imaging®, Ibex® EVO®; Loveland, Colorado, USA) with a 6.4 MHz CL3E transducer placed on the plastron. Heart beats were counted for 15 s and then multiplied by 4 to determine beats/min.

Whole blood was then collected from the external jugular vein using 1 ml 26-gauge BD allergy syringes (Becton, Dickinson and Co., Franklin Lakes, New Jersey, USA) and safe blood collection practices for reptiles as outlined by Stacy and Innis (2017) and NOAA SEFSC (2008). Baseline blood analytes included whole blood glucose, total solids, PCV, hemoglobin and hemogram variables (e.g. total WBC count, WBC differential, red and WBC morphology) as outlined below.

After sample collection, whole blood samples were gently inverted to ensure adequate mixing of blood with anticoagulant and immediately analyzed for glucose using an EasyTouch® glucose monitoring system (MHC® Medical Products, Fairfield, Ohio, USA) based on glucose oxidase and potentiometry with test strips for use with capillary whole blood. The test strips were adequately filled with whole blood per the manufacturer’s recommendations. Calibrations of the instrument were also performed before each series of analyses per manufacturer’s recommendations. This system has been validated for determining blood glucose concentrations in green and loggerhead sea turtles (Perrault *et al*., 2018; Kunze *et al*., 2020).

Next, we determined PCV from whole blood collected into a microcapillary tube (Fisher Health-Care, Houston, Texas, USA) with Critoseal® (Sherwood Medical Co., Deland, Florida, USA) as the sealant. The capillary tubes were spun for 5 min at 1300 *g* (5000 rpm) using a ZipCombo microhematocrit centrifuge (LW Scientific, Inc., Lawrenceville, Georgia, USA). A hematocrit microcapillary tube reader was used to determine PCV as a percentage. The plasma color above the red blood cell (RBC) column was evaluated for plasma discoloration (e.g. hemolysis). The capillary tubes were then broken above the buffy coat, and plasma from these tubes was used to obtain total solids using a Reichert VET 360 handheld refractometer (Reichert Technologies Analytical Instruments, Depew, New York, USA).

Hemoglobin concentrations in whole blood were analyzed using a portable hemoglobinometer (HemoCue® 201+ analyzer, HemoCue Inc., Lake Forest, California, USA). The blood sample was drawn into a cuvette by capillary action, where the hemoglobin was released after disintegration of erythrocyte membranes by sodium deoxycholate. The hemoglobin iron was then oxidized from the ferrous to the ferric state by sodium nitrite to form methemoglobin and then combined with azide to form azidemethemoglobin (HemoCue Inc., 2016; Stacy *et al*., 2019).

Blood films were prepared from well-mixed whole blood, air-dried and stained with Wright-Giemsa (Harleco®, EMD Millipore, Billerica, Massachusetts, USA). Blood film evaluation was performed by one co-investigator (NIS) and included WBC estimate (Weiss 1984), a 200 WBC differential (including heterophils, lymphocytes, monocytes, eosinophils, basophils) and morphological evaluation of RBCs, WBCs and thrombocytes. The heterophil/lymphocyte ratio was calculated.

### Locomotor performance

Following blood collection, hatchlings were subjected to the self-righting performance test. A 19-L bucket was filled halfway with seawater. Each hatchling was placed upside down on its carapace at the surface of the seawater. The time it took the hatchlings to right themselves was recorded using a stopwatch to the nearest 0.01 s and then repeated three times for an average. Once sample collection was complete, all hatchlings were released into the ocean the same night on Juno Beach after a brief observation period to ensure all hatchlings were behaving normally and fit for release.

### Statistical analyses

Data were pooled by month and tested for normality using the Shapiro–Wilk test. Given the violation of the assumptions of normality, nonparametric Kruskal–Wallis tests were performed to examine differences in monthly averages of incubation temperature, incubation duration, hatching morphology and locomotor performance. If the results of the Kruskal–Wallis were significant, Bonferroni post hoc tests were also performed to examine which monthly differences were significant. Outliers that were outside 1.5 * IQR (interquartile range), where IQR is the difference between the 25th and 75th quartiles, were removed.

Additionally, linear regressions were performed to test the association between continuous temperature data (T_inc_) and morphology, health indices and locomotor performance. Any PCV, total solids, and hemoglobin values with associated hemolysis scores of 2+ were removed from both Kruskal–Wallis tests and linear regressions (Stacy and Innis, 2017). Linear regressions were also performed to test for an association between righting time, hemoglobin and heart rate as well as PCV and hemoglobin. Furthermore, a multiple linear regression was used to predict locomotor performance based on morphological data including SCL_min_, SCW, BD, FFL and mass. Assumptions of the regressions were assessed using diagnostic plots and the Shapiro–Wilk test on residuals. Variables were log transformed to comply with the assumptions of normality.

Reference intervals for whole blood glucose concentrations, PCV, hemoglobin concentrations, total solids and leukogram variables were calculated after Friedrichs *et al*. (2012) using nonparametric methods for sample sizes ≥120. Samples with hemolysis scores of ≥2+ were eliminated prior to calculating reference intervals for PCV, total solids, glucose and hemoglobin (Stacy *et al*., 2019).

Hatchling SCL_min_ was positively correlated with SCW, FFL, mass and heart rate and negatively correlated with righting response time, hemoglobin, total solids, PCV and umbilicus length and width; thus, we divided the variables by the hatchling’s SCL_min_ to ensure observed differences were not due to size. Statistical significance was determined if *P* ≤ 0.05. All statistical tests were performed in R 3.6.1 using packages ‘FSA’, ‘dplyr’, ‘car’ and ‘ggplot2’ (R Core Team 2019).

## Results

### Hatchlings

All hatchlings appeared and behaved clinically normal for the species and life-stage class during the evaluation period of each turtle. The turtles were active and alert and did not exhibit any overt external abnormalities.

### Incubation temperatures

**Figure 2:**
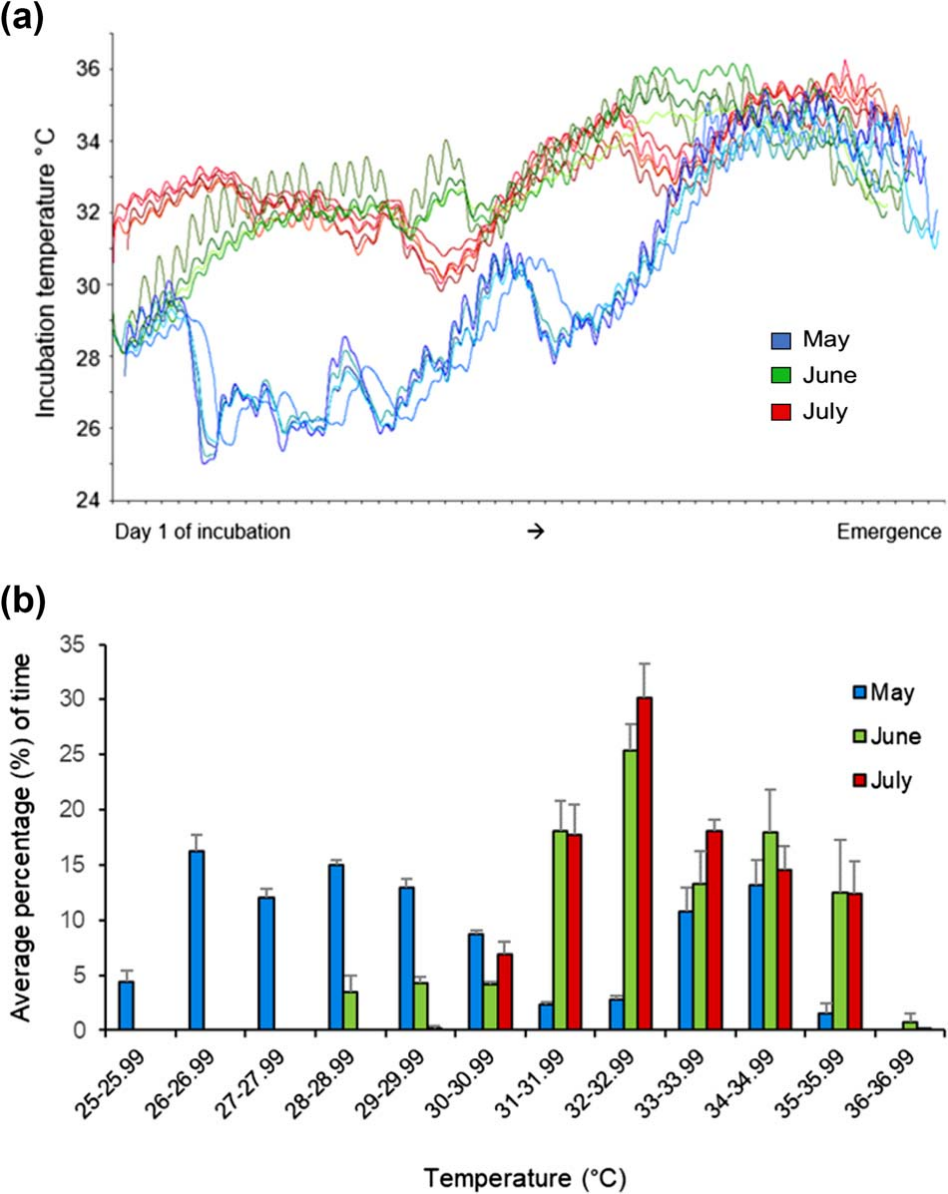
Temperature data for 14 loggerhead sea turtle (*C. caretta*) nests observed during May through July 2018 at Juno Beach. (**a**) Transposed temperature data for 14 nests obtained from the data loggers demonstrate monthly variation. Incubation temperatures (°C) throughout the entire incubation period are portrayed on the y-axis with the start of the x-axis representing Day 1 of incubation for all nests and continuing through to hatch out. (**b**) Monthly variation in the amount of time spent in each 1°C range, with the average percentage of time portrayed on the y-axis and the 1°C temperature ranges portrayed on the x-axis

**Figure 3:**
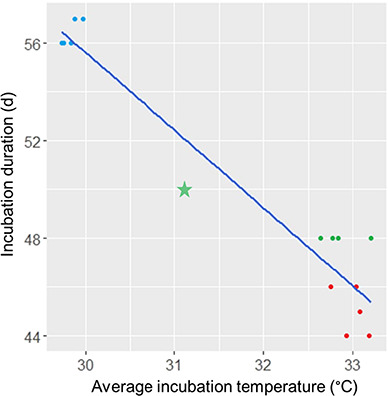
Loggerhead sea turtle (*C. caretta*) incubation duration in relation to average nest incubation temperatures as observed during May through July 2018 at Juno Beach. Incubation duration (in days) for each nest is represented on the y-axis with the average *in situ* incubation temperature portrayed on the x-axis. Blue circles correspond to nests laid in May, green circles correspond to nests laid in June and red circles correspond to nests laid in July. The green star in the middle of the graph represents the inundated June nest that was removed from morphological and physiological statistical analyses. The regression line was statistically significant (y = −3.22x + 152.09; *R^2^* = 0.93*, P* < 0.001)

Temperature data for 14 nests (May = 5, June = 4, July = 5) obtained from the data loggers are shown in Fig. 2a. The amount of time the nests in May, June and July were in each 1°C range is also shown in Fig. 2b. One nest in June was inundated by the high tide on 4 consecutive days (10–13 July). Hatchlings from that nest were collected and sampled similar to the other clutches, but all results from that nest were excluded from analyses due to a number of the results, particularly with temperature and health analyses, being outliers. The relationship between incubation temperature and incubation duration is shown in Fig. 3, where the June outlier nest is clearly shown in the center of the graph. Incubation duration was significantly correlated with incubation temperature (*R*^2^ = 0.93, *P* < 0.001). The overall incubation temperature, T_inc_, (*χ^2^ (2)* = 8060.1, *P* < 0.001), incubation temperature during the middle third of incubation, T_mid_, (*χ^2^ (2)* = 7987.6, *P* < 0.001) and during the last 2 weeks of incubation, T_2w_, (*χ^2^ (2)* = 1064.9, *P* < 0.001) were significantly different in May, June and July. The 3-day maximum temperature, T_3dm,_ (*χ^2^ (2)* = 868.2, *P* < 0.001), was significantly lower in May than in June and July. The incubation period (*χ^2^ (2)* = 12.1, *P* = 0.002) was significantly longer in May than in June and July. Temperatures, incubation period and post-hoc results are reported in Table 1. All nests (14/14) experienced a mean T_3dm_ of >34°C, with 8/14 (57%) nests experiencing a T_3dm_ of over 35°C; 2/14 (14%) nests experienced temperatures over 36°C, but only for several hours (Fig. 2b).

### Morphometrics

#### Hatchling size

Results from all morphological analyses are presented in Table 2. Hatchlings from nests laid in May were the longest compared to the other months (SCL_min_: *χ^2^ (2)* = 50.0, *P* < 0.001; May v June: *P* < 0.001; May v July: *P* < 0.001; June v July: *P* = 0.519). The same occurred with hatchling width (SCW: *χ^2^ (2)* = 21.3, *P* < 0.001; May v June: *P* < 0.001; May v July: *P* < 0.001; June v July: *P* = 0.812). In contrast, May’s clutches had hatchlings with smaller BD than June and July (BD: *χ^2^ (2)* = 43.9, *P* < 0.001; May v June: *P* < 0.001; May v July: *P* < 0.001; June v July: *P* = 0.558). FFL was significantly different (*χ^2^ (2)* = 31.6, *P* < 0.001) between all months with hatchlings from May having the longest flipper length, followed by July, then June (May v June: *P* < 0.001; May v July: *P* = 0.009; June v July: *P* = 0.005). Mass varied significantly by month (*χ^2^ (2)* = 15.4, *P* < 0.001), with May nests producing heavier hatchlings in comparison to hatchlings produced in June (May v June: *P* < 0.001); however, mass from May and June nests did not differ statistically from those laid in July (May v July: *P* = 0.173; June v July: *P* = 0.078). Results from the CSI and BCI calculations indicate that hatchlings from May nests had the largest CSI (*χ^2^ (2)* = 42.2, *P* < 0.001) but smallest BCI (*χ^2^ (2)* = 50.3, *P* < 0.001). CSI was significantly higher in May compared to June and July (May v June: *P* < 0.001; May v July: *P* < 0.001; June v July: *P* = 0.662); however, BCI was significantly lower in May than in June and July (May v June: *P* < 0.001; May v July: *P* < 0.001; June v July: *P* = 0.598). Hatchling SCL_min_ was positively correlated with several variables, including SCW (*R*^2^ = 0.41, *P* < 0.001), FFL (*R*^2^ = 0.34, *P* < 0.001) and mass (*R*^2^ = 0.38, *P* < 0.001).

**Table 1:** Summary of *in situ* incubation temperatures for loggerhead sea turtle (*C. caretta*) nests in May through July 2018 at Juno Beach. Data are represented in terms of mean ± standard error (SE). Median and range are also reported. Different superscript letters indicate significant differences between months

		May	June	July	*P-*value
T_inc_ (°C)	Mean ± SE	29.8^a^ ± 0.03	32.9^b^ ± 0.02	33.0^c^ ± 0.01	*P* < 0.001
	Median	29.1	32.7	32.8	
	Range	25.0–35.5	27.9–36.2	29.8–36.3	
T_mid_ (°C)	Mean ± SE	28.8^a^ ± 0.02	32.8^b^ ± 0.02	32.3^c^ ± 0.02	*P* < 0.001
	Median	28.9	32.6	32.2	
	Range	25.9–31.2	31.2–35.7	29.8–34.9	
T_2w_ (°C)	Mean ± SE	34.1^a^ ± 0.01	34.6^b^ ± 0.02	34.5^c^ ± 0.01	*P* < 0.001
	Median	34.1	34.8	34.7	
	Range	32.2–35.5	32.2–36.2	32.2–36.3	
T_3dm_ (°C)	Mean ± SE	34.6^a^ ± 0.02	35.3^b^ ± 0.02	35.3^b^ ± 0.01	*P* < 0.001
	Median	34.6	35.3	35.3	
	Range	33.8–35.5	34.6–36.2	34.3–36.3	
Incubation period (d)	Mean ± SE	56^a^ ± 0.2	48 ^ab^ ± 0	45 ^b^ ± 0.5	*P* = 0.002
	Median	56	48	45	
	Range	56–57	48	44–46	

Abbreviations and statistical results: T_inc_, temperature during the entire incubation period (May v June: *P* < 0.001; May v July: *P* < 0.001; June v July: *P* = 0.027); T_mid_, temperature in the middle third of incubation (May v June: *P* < 0.001; May v July: *P* < 0.001; June v July: *P* < 0.001).; T_2w_, temperature during the last 2 weeks of incubation (May v June: *P* < 0.001; May v July: *P* < 0.001; June v July: *P* < 0.001); T_3dm_, 3-day maximum temperature (May v June: *P* < 0.001; May v July: *P* < 0.001; June v July: *P* = 0.495); incubation period (May v June: *P* = 0.303; May v July: *P* = 0.002; June v July: *P* = 0.303).

**Table 2:** Monthly variation of loggerhead sea turtle (*C. caretta*) hatchling morphology observed during May through July 2018 at Juno Beach. Data are represented in terms of mean ± SE. Median, range and sample size are also reported. Different superscript letters indicate significant differences between months

		May	June	July	*P*-value
SCL_min_ (mm)	Mean ± SE	43.7^a^ ± 0.1	41.3^b^ ± 0.3	42.0^b^ ± 0.2	*P* < 0.001
	Median	43.8	41.5	42.2	
	Range	41.7–45.6	36.9–44.7	39.1–44.6	
	*N*	49	34	50	
SCW (mm)	Mean ± SE	32.5^a^ ± 0.1	30.9^b^ ± 0.3	31.7^b^ ± 0.2	*P* < 0.001
	Median	32.5	31.5	31.9	
	Range	30.0–33.9	26.2–33.7	29.0–34.1	
	*N*	49	33	50	
BD (mm)	Mean ± SE	17.8^a^ ± 0.1	18.9^b^ ± 0.1	18.8^b^ ± 0.1	*P* < 0.001
	Median	17.8	18.9	18.8	
	Range	16.3–19.2	17.7–19.6	16.9–21.1	
	*N*	49	30	50	
FFL (mm)	Mean ± SE	36.6^a^ ± 0.2	33.6^b^ ± 0.4	35.1^c^ ± 0.2	*P* < 0.001
	Median	36.2	33.4	35.1	
	Range	32.8–37.5	29.9–37.6	32.2–38.1	
	*N*	37	34	50	
Mass (g)	Mean ± SE	18.4^a^ ± 0.1	17.1^b^ ± 0.2	17.9^ab^ ± 0.2	*P* < 0.001
	Median	18.2	17.0	18.0	
	Range	16.5–20.1	14.5–19.6	14.4–21.4	
	*N*	49	34	50	
CSI (mm^2^)	Mean ± SE	1420^a^ ± 8	1271^b^ ± 24	1332^b^ ± 11	*P* < 0.001
	Median	1432	1313	1341	
	Range	1269–1510	915–1472	1156–1500	
	*N*	49	34	50	
BCI (g/mm^3^)	Mean ± SE	2.15^a^ ± 0.01	2.41^b^ ± 0.03	2.41^b^ ± 0.03	*P* < 0.001
	Median	2.17	2.39	2.36	
	Range	1.91–2.27	2.13–2.84	2.09–2.99	
	*N*	37	34	50	

Abbreviations: SCL_min_, minimum straight carapace length; SCW, straight carapace width; BD, body depth; FFL, front (right) flipper length; CSI, carapace size index; BCI, body condition index.

Temperature was negatively associated with SCL_min_ (*R*^2^ = 0.37, *P* < 0.001), SCW (*R*^2^ = 0.16, *P* < 0.001) and CSI (*R*^2^ = 0.31, *P* < 0.001) and positively associated with BD (*R*^2^ = 0.49, *P* < 0.001) and BCI (*R*^2^ = 0.23, *P* < 0.001).

#### Umbilical scar length and width

There was a significant difference between months for both umbilical scar length (*χ^2^ (2)* = 42.3, *P* < 0.001) and width (*χ^2^ (2)* = 33.5, *P* < 0.001). Umbilical scar length was smaller in hatchlings from nests laid in May (6.1 mm ± 0.1 mm), compared to hatchlings from nests laid in June (7.1 mm ± 0.2 mm) and July (7.5 mm ± 0.2 mm) (May v June: *P* < 0.001; May v July: *P* < 0.001; June v July: *P* = 0.858). A similar trend was observed for umbilical scar width with hatchlings from May (4.5 mm ± 0.1 mm) having a non-significantly smaller width than hatchlings from June nests (4.7 mm ± 0.2 mm) and a significantly smaller width than July hatchlings (5.6 mm ± 0.2 mm) (May v June: *P* = 0.062; May v July: *P* < 0.001; June v July: *P* = 0.105). Hatchling SCL_min_ negatively correlated with umbilicus length (*R*^2^ = 0.11, *P* < 0.001) and umbilicus width (*R*^2^ = 0.07, *P* = 0.002).

**Figure 4:**
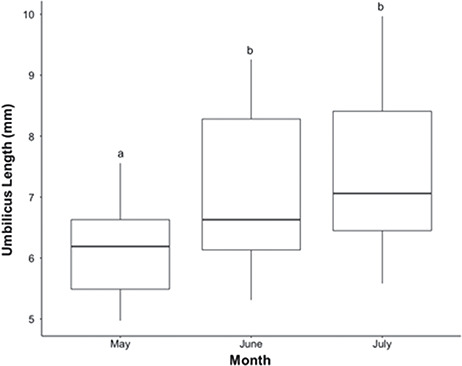
Differences in umbilical scar length of loggerhead sea turtle (*C. caretta*) hatchlings by month as observed during May through July 2018 at Juno Beach. Boxplots show the median (solid line within box), 1st and 3rd quartiles (lower and upper box limits) and range (whiskers). Different lowercase letters represent statistically significant differences between months

Average incubation temperature was positively associated with umbilical scar length (*R*^2^ = 0.34, *P* < 0.001; Fig. 4) and width (*R*^2^ = 0.17, *P* < 0.001).

### Health variables

Measures of central tendency, range and reference intervals for glucose, total solids, PCV, hemoglobin, leukogram variables and heart rate are shown in Table 3, with monthly variation of these variables shown in **Supplemental Table 1**. Ten data points for glucose, total solids, PCV and hemoglobin measurements were eliminated from statistical analyses due to the samples having a hemolysis score of **≥**2+ (Stacy and Innis, 2017; Stacy *et al*., 2019).

**Table 3:** Measures of central tendency, range and reference intervals for whole blood glucose, total solids, PCV, hemoglobin, leukogram variables and heart rate for loggerhead sea turtle (*C. caretta*) hatchlings from Juno Beach. Nonparametric methods for sample sizes ≥120 were used to calculate reference intervals after Friedrichs *et al*. (2012). For glucose, total solids, PCV and hemoglobin, samples with a hemolysis scores ≥2+ were eliminated during calculation of reference intervals

Parameter	MEAN ± SD	Median	Range	*n*	Lower limit (90% CI)	Upper limit (90% CI)
Glucose [mmol/l]	5.1 ± 1.2	5.1	2.2–8.4	130	3.1 (2.2–3.3)	7.4 (7.1–8.4)
Total solids [g/l]	32 ± 5	32	20–59^a^	132	23 (20–25)	42 (40–44)
PCV [%]	34 ± 6	34	23–46	128	24 (23–26)	45 (44–46)
Hemoglobin (g/l)	86 ± 11	87	60–109	130	65 (60–70)	106 (103–109)
Total WBC [×10^3^/μl]	8.6 ± 2.4	8.1	4.7–14.1	134	4.9 (4.7–5.2)	13.7 (12.8–14.1)
Absolute heterophils [×10^3^/μl]	4.7 ± 1.6	4.3	2.2–9.3	134	2.4 (2.2–2.6)	8.6 (7.8–9.3)
Absolute immature heterophils [×10^3^/μl]	0.1 ± 0.2	0.1	0–0.8	134	0 (0)	0.7 (0.4–0.8)
Absolute lymphocytes [×10^3^/μl]	2.7 ± 0.9	2.5	0.8–5.0	134	1.2 (0.8–1.5)	4.8 (4.4–5.0)
Absolute monocytes [×10^3^/μl]	0.8 ± 0.5	0.7	0.2–2.8	134	0.2 (0.2–0.3)	2.3 (1.8–2.8)
Absolute eosinophils [×10^3^/μl]	0	0	0	134	0 (0)	0 (0)
Absolute basophils [×10^3^/μl]	0.3 ± 0.5	0.2	0–3.6	134	0.1 (0–0.1)	2.0 (0.7–3.6)
Heterophil/lymphocyte	1.90 ± 0.77	1.77	0.81–5.55	134	0.86 (0.81–1.00)	3.87 (3.12–5.55)
Heart rate (beats/min)	77 ± 8	76	60–98	142	62 (60–68)	94 (90–98)
^a^ 59 g/l is an outlier. The next highest value was 44 g/l. Reference intervals were calculated with this value removed.						

Incubation temperature was significantly and positively associated with total solids (*R*^2^ = 0.22, *P* < 0.001), PCV (*R*^2^ = 0.04, *P* = 0.023), hemoglobin (*R*^2^ = 0.18, *P* < 0.001), total WBC count (*R*^2^ = 0.05, *P* = 0.007), absolute heterophils (*R*^2^ = 0.07, *P* = 0.002), absolute basophils (*R*^2^ = 0.05, *P*= 0.012) and the heterophil/lymphocyte ratio (*R*^2^ = 0.10, *P* < 0.001). PCV had a significant correlation with hemoglobin (*R*^2^ = 0.55, *P* < 0.001), resulting in an equation of Hbg [g/l] = (PCV × 1.39) + 38.48. Simply, hemoglobin for loggerhead hatchlings is ~2.5X their PCV when using g/l, the SI unit.

**Figure 5:**
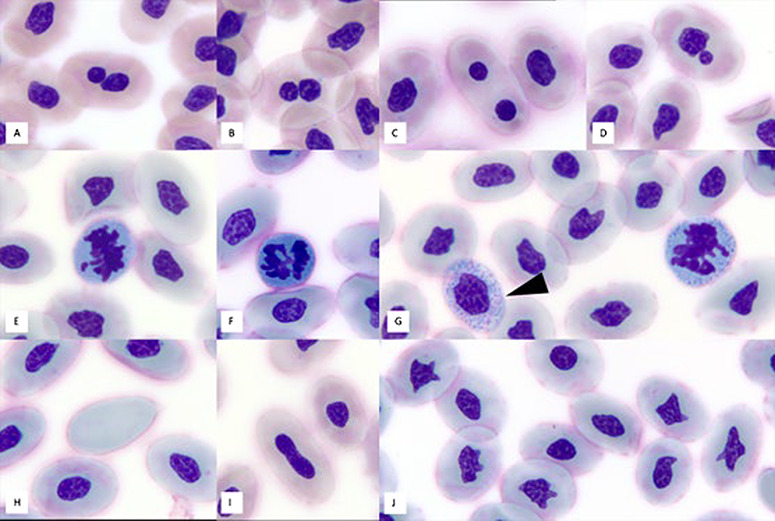
Representative images of erythrocytes in loggerhead sea turtle (*C. caretta*) hatchlings from Juno Beach showing binucleation (**A**–**C**), a micronucleus (**D**), mitotic figures (**E**–**G**), basophilic stippling (arrowhead), an erythroplastid (**H**) and nuclear dysplasia (**I**–**J**). ×100 objective, Wright–Giemsa stain

**Figure 6:**
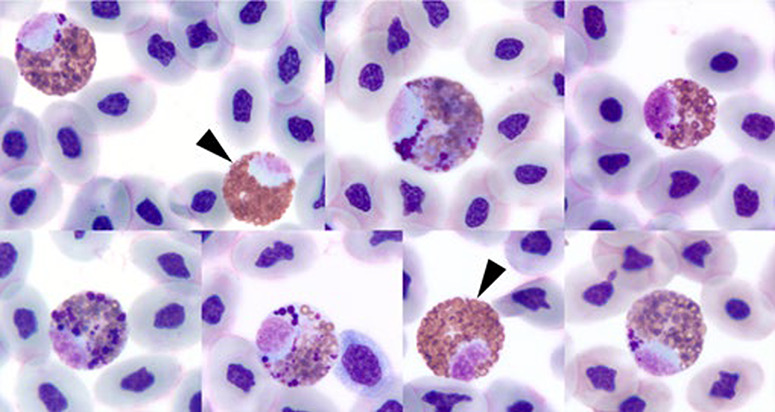
Heterophils of loggerhead sea turtle (*C. caretta*) hatchlings from Juno Beach showing representative images of immature heterophils with primary granules in comparison to two mature heterophils (arrowheads). ×100 objective, Wright–Giemsa stain.

There were also significantly more mitotic figures in immature RBCs (*χ^2^ (2)* = 58.0, *P* < 0.001) in July compared to May and June (May v June: *P* = 0.766; May v July: *P* < 0.001; June v July: *P* < 0.001). RBCs observed throughout the study period had mild nuclear dysplasia visible as micronuclei, binucleation or pleomorphism; there was also minimal basophilic stippling and rare erythroplastids; very low numbers of immature heterophils were observed in most hatchlings (Figs 5, 6).

Hatchling SCL_min_ was negatively correlated with total solids (*R*^2^ = 0.10, *P* = 0.002), PCV (*R*^2^ = 0.09, *P* = 0.002) and hemoglobin (*R*^2^ = 0.04, *P* = 0.016).

Hatchling heart rate was significantly lower in July (73 bpm ± 1 bpm) compared to May (81 bpm ± 1 bpm) and June (78 bpm ± 1 bpm; *χ^2^ (2)* = 20.2, *P* < 0.001), with hatchlings from May having the highest rate and hatchlings from July having the lowest rate (May v June: *P* = 0.449; May v July: *P* < 0.001; June v July: *P* = 0.006). Heart rate was also negatively associated with incubation temperature (*R*^2^ = 0.11, *P* < 0.001) and positively associated with SCL_min_ (*R*^2^ = 0.04, *P* = 0.01).

### Locomotor performance

**Figure 7:**
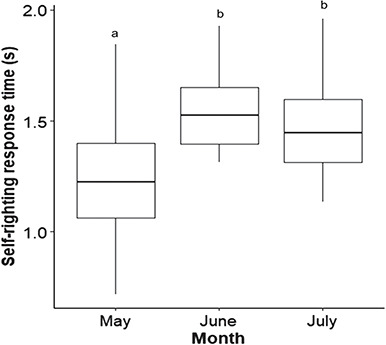
Differences in self-righting response (seconds) of loggerhead sea turtle (*C. caretta*) hatchlings by month as observed during May through July 2018 at Juno Beach. Boxplots show the median (solid line within box), 1st and 3rd quartiles (lower and upper box limits) and range (whiskers). Different lowercase letters represent statistically significant differences between months

There was a significant difference in self-righting response time (*χ^2^ (2)* = 33.3, *P* < 0.001, Fig. 7) with righting time fastest in May (May v June: *P* < 0.001; May v July: *P* < 0.001; June v July: *P* = 0.836). A multiple regression was calculated to predict response time based on morphological data including SCL_min_, SCW, BD, FFL and mass. The regression was significant (*R*^2^ = 0.28, *P* < 0.001) with the only predictor of righting response being SCL_min_*(P* = 0.004). In addition, self-righting time was negatively associated with incubation temperature (*R*^2^ = 0.27, *P* < 0.001) and SCL_min_ (*R*^2^ = 0.18, *P* < 0.001). Lastly, hemoglobin was positively associated with self-righting time (*R*^2^ = 0.11, *P* < 0.001), but self-righting response time was not significantly related to heart rate (*P* > 0.05).

## Discussion

Understanding and attempting to outline predictive models for how wildlife species will respond to climate change is imperative when developing species management and recovery plans. This study investigated how the month of hatching influences morphometrical and physiological traits in loggerhead hatchlings; it also provides critical information regarding *in situ* nest temperatures on Juno Beach, Florida, USA, which represents one of the most densely nested loggerhead beaches in the world (LMC, unpublished data). This study is the first comprehensive investigation regarding the effects of *in situ* incubation temperatures on various physical and physiological characteristics of sea turtle hatchlings. Generally, we found hatchlings coming from warmer nests were associated with smaller body size and higher BCI, larger umbilical scar size, slower righting time, lower heart rates, altered hemodynamic balance (e.g. dehydration) and potential inflammation and/or stress, as discussed in detail below. These findings provide important insights into potential impacts of rising environmental temperatures on the development of loggerhead sea turtle embryos and hatchlings *in situ*, suggesting increasing risks of developing suboptimal physiological features affecting overall fitness and ultimately survival.

### *In situ* nest temperatures

Nest temperatures recorded during this study provide unique information regarding *in situ* incubation temperatures of loggerhead sea turtles nesting on Florida’s beaches. Obtaining data from *in situ* nests is important, since laboratory experiments do not mimic natural nest environmental conditions due to differences in natural variability (e.g. humidity, diurnal temperature variations), water uptake, gas exchange and metabolic heating (Horne *et al*., 2014; Booth, 2017). This makes the data obtained from *in situ* studies critical, especially for this very important nesting population, which has recently been considered an area of concern for decreased hatching success due to predicted warming (Monsinjon *et al*., 2019). Since the current estimated lethal threshold for constant incubation temperatures is between 33°C and 35°C (Howard *et al*., 2014), it is of possible concern that the majority of nests from this study were exposed to temperatures within this range, specifically, two nests experienced temperatures above 36°C, while 14/15 (93%) of nests experienced a mean 3-day maximum temperature (T_3dm_) of over 34°C and 8/15 (53%) experienced a T_3dm_ of over 35°C. It is not uncommon for natural, temperature-variable nests in Florida to reach these high temperatures, especially at the end of incubation and during the mid- to late-nesting season. However, the fact that some of these temperatures were recorded in May, the typically cooler portion of the loggerhead nesting season and that they remained in this range for at least three consecutive days, is a potential area of concern. Additionally, spending 30–39% of the incubation period in these high temperature ranges (34–35.5°C) has been shown to significantly increase embryonic mortality in Florida (Bladow and Milton, 2019). Under an optimistic warming scenario (radiative forcing that will stabilize at +4.5 W.m^−2^ compared to preindustrial values), hatching success at Boca Raton, Florida, USA by 2100 (in southern Palm Beach County) is expected to be lower than 20%, with success dipping below 10% under a pessimistic scenario (radiative forcing that will reach +8.5 W.m^−2^ by 2100; Monsinjon *et al*., 2019). Although temperatures in June and July were similar in this study, the amount of time the nests in these 2 months were exposed to these temperatures varied, with nests in June spending 7.5% more time in the 28–29.99°C range and 3.4% more time in the 34–34.99°C range, while nests in July spent 9.5% more time in the 32–33.99°C range. The differences in the amount of time nests in June and July experienced certain temperatures could explain some of the resulting differences seen between the 2 months, specifically in regards to health variables. Future studies should examine loggerhead nest temperatures in April on Juno Beach to determine if incubation temperatures are significantly cooler than May and if there are possible correlations to significantly different results in morphology, health variables and performance.

### Warmer nests produce smaller hatchlings

Our results support previous laboratory and field-based studies, whereby increased incubation temperatures resulted in smaller hatchlings (Glen *et al*., 2003; Mickelson and Downie, 2010; Read *et al*., 2012; Sim *et al*., 2015). BCI was the lowest in May. For juveniles and adults, a larger BCI score is thought to correspond to a healthier individual; however, no studies to our knowledge analyze this association in hatchlings. Various BCI scoring systems exist, which may yield different results, showcasing the need for a universal BCI for hatchlings. The BCI equation for immature and mature life-stage classes provided by Bjorndal *et al*. (2000) may not be the best indicator of quality for the hatchling life-stage class since yolk mass constitutes a large proportion of body mass at this stage. A different equation by Nagy *et al*. (2002) that takes into consideration mass, length, width and body depth, might be a better fit for sea turtle hatchlings, especially since the authors report that it can be a good first estimate of hydration. Therefore, future research should examine these varying BCI scoring systems to establish a universal index for hatchlings. However, it should be noted that we cannot rule out that differences in size were not impacted by moisture levels or parental origin since both significantly influence hatchling phenotype and were not tested in this study (Booth *et al*., 2012).

Longer incubation periods in sea turtles lead to larger hatchlings at time of emergence. The larger sizes of hatchlings produced in May might be advantageous in avoiding predation, since many predators that hatchlings encounter are gape limited (Gyuris, 2000; Janzen *et al*., 2000; Booth, 2006; Salmon and Scholl, 2014; Salmon *et al*., 2015). Conversely, umbilical scar length and width were smaller in May compared to June and July. Umbilicus scar size significantly correlates with the amount of yolk reserve in loggerhead and green turtle hatchlings (Perrault, unpublished data); therefore, this study supports previous studies, whereby cooler nests produce hatchlings that have converted more yolk material to tissue, thus having smaller residual yolk remaining after hatching (Booth, 2000; Booth and Astill, 2001; Hewavisenthi and Parmenter, 2001; Glen *et al*., 2003; Booth and Evans, 2011). Hatchlings must rely on these yolk reserves for the metabolic demands of hatching, emergence, trek from nest to open ocean and during the early stages of their offshore frenzy and migration (Kraemer and Bennett, 1981; Salmon and Wyneken, 1987; Jones *et al*., 2007). When incubated between 28–30°C, green turtle hatchlings should be able to survive for at least 10 days using their yolk reserves before needing to feed (Booth, 2009). Thus, hatchlings from warmer nests that have larger yolk reserves are more likely to survive longer without eating. This larger portion of yolk to body mass might also lead to hatchlings in warmer nests having a higher buoyancy than those from cooler nests, which could then result in differences in energy expenditure in regards to swimming. However, hatchlings with smaller yolk reserves may be at risk of depleting their yolk supply before they reach their refuge habitat, possibly resulting in short-term starvation and subsequently a catabolic state of metabolism (March *et al*., 2019), again with higher risk of exposure to predators.

### Warmer nests lead to hatchlings with altered hemodynamic balance

In loggerhead hatchlings from this study, total solids, PCV and hemoglobin were concurrently higher in warmer months (June or July) when compared to the cooler month (May), suggesting altered hemodynamic balance through shifts in hydration status (i.e. early stage dehydration). PCV represents the proportion of RBCs in comparison to total volume of blood and serves as a useful indicator of dehydration when elevated or of anemia when low (Stacy and Innis, 2017). It has also been found to vary with water temperature and life-stage class (Stamper *et al.,* 2005; Kelly *et al*., 2015; Perrault *et al*., 2016; Stacy and Innis, 2017). The PCV data reported in loggerhead hatchlings from this study (mean, 34%; range, 23–46%) is comparatively higher than those reported for loggerhead hatchlings from North Carolina (Harms *et al*., 2019: mean, 25%; range, 19–32%), green turtle post-hatchlings from Surinam (Frair, 1977: mean, 29%; range, 27–33%), green turtle hatchlings from Heron Island, Australia (Wells and Baldwin, 1994: mean, 28%), post-hatchling green turtles from Heron Island that underwent short-term fasting for 3–15 h (March *et al*., 2019: range, 13–27%), juvenile loggerheads from the Azores (Stacy *et al*., 2018: mean, 22%; range, 14–32%), 8–56-month-old captive reared loggerheads from Florida (Rousselet *et al*., 2013: range, 17–32%) and leatherback hatchlings from Costa Rica (Reina *et al*., 2002: mean, 30%). Increased PCV can result from dehydration in leatherback hatchlings (Reina *et al*., 2002); therefore, it is possible that higher incubation temperatures could cause dehydration and thus increased PCV.

Similar to PCV, hemoglobin concentrations in loggerhead hatchlings from our study were also found to be positively associated with incubation temperatures. Hemoglobin is the oxygen-transporting protein in RBCs (Wells and Baldwin, 1994) and can also indicate dehydration or anemia as it is an indirect measurement of PCV (Stacy *et al.*, 2019). Previous studies on leatherbacks (Stacy *et al.*, 2019) and several avian orders (Velguth *et al*., 2010) found significant correlations between PCV and hemoglobin concentrations, similar to our results, which show that hemoglobin for loggerhead hatchlings is ~2.5X their PCV when using SI units. The observation of mild nuclear dysplasia, micronuclei, binucleation, pleomorphism, basophilic stippling and presence of rare erythroplastids suggest normal RBC morphological features of loggerhead sea turtle hatchlings.

Total solids provide information primarily regarding the amount of proteins (also lipids and electrolytes) in plasma (Bolten and Bjorndal, 1992), indicating dehydration and/or inflammation when elevated, or various conditions of protein loss or decreased production or absorption when low (Stacy and Innis, 2017). As with PCV and hemoglobin, plasma total solids in loggerhead hatchlings from this study were also positively associated with incubation temperatures. One consideration for higher total solids in warmer months includes possible higher absorption of yolk constituents (e.g. proteins, lipids) due to increased metabolic rates in warmer months. The mean concentration of total solids from loggerhead hatchlings this study (32 g/l) is also higher than that reported for 8–56-month-old (range, 9–20 g/l) captive-reared loggerheads from Florida (Rousselet *et al*., 2013). Hatchling size (SCL_min_) in this study negatively correlated with total solids, PCV and hemoglobin. Since little is known about baseline health indices for hatchlings immediately following emergence, hatchling size as a driver for these indices has yet to be investigated.

The higher number of mitotic figures observed in immature RBCs in hatchlings from warmer nests signifies an increased release of immature RBC stages from hematopoietic tissues, which is common in young reptiles (Campbell, 1996). However, increased erythropoiesis has been associated with reduced oxygen availability or increased oxygen demand, which is a plausible consideration for hatchlings from warmer nests, since dehydration can result in slower blood flow, increased blood viscosity and thus reduced oxygen availability in circulating blood (Stone *et al*., 1968). Furthermore, increased oxygen demand in warmer nests may result from higher metabolic rates and increased oxygen consumption in warmer nests, consequently resulting in reduced oxygen availability within the nest. Therefore, the concomitant increases in PCV, hemoglobin, total solids and the decrease in heart rate provide evidence that higher nest incubation temperatures could be related to altered hydration status and/or other hemodynamic derangements in loggerhead hatchlings upon emergence. These impacts could lead to earlier exhaustion and decreased overall fitness and locomotor performance, potentially impacting early sea turtle life-stage class survival.

WBC concentration, absolute heterophils, absolute basophils and the heterophil/lymphocyte ratio were positively correlated with incubation temperatures. Peripheral WBC concentrations are considered useful diagnostic indicators of inflammation or stress (Stacy and Innis, 2017) and have been observed to be influenced by water temperature (Kelly *et al*., 2015). This is the first study to examine baseline health variables of sea turtle hatchlings and associations with nest temperatures. Additional studies are needed to better establish baselines for not only loggerhead hatchlings but also for hatchlings of other sea turtle species across geographical locations. Understanding the impacts of incubation temperatures on sea turtle early life-stage class immunity is important, as increases in total WBC counts or variations in WBC differentials could suggest potential underlying systemic inflammation, immune stimulation, stress or other less frequently considered conditions (Stacy and Innis, 2017). The observed concurrently higher heterophils and basophils suggest the presence of inflammation in hatchlings incubated in warmer nests rather than stress. Twenty-eight-day-old loggerhead hatchlings from North Carolina that incubated at temperatures ranging from 27.2–30.8°C had lower WBC estimates, and fewer basophils and lymphocytes (Harms *et al*., 2019) compared to the hatchlings from this study, indicative of inflammation in the hatchlings from warmer nests in this study. Inflammation is further suggested by the presence of immature heterophils, which were frequently observed across study turtles, although not significantly correlated with incubation temperatures. The positive correlation of the heterophil/lymphocyte ratio with nest incubation temperature suggests stress as an additional factor affecting hatchlings incubated in warmer nests. Behavioral stress of hatchlings from this study was not overt during the examination period; however, stress from handling/during sampling cannot definitely be ruled out as a factor impacting hematology results, but is considered less likely to have had an influence on study hatchlings since the hatchlings throughout the study period were treated similarly and behaved normally after emergence, transport and sampling.

Heart rate was significantly lower in July than in May and June and was significantly associated with average incubation temperature. Embryonic heart rates have been found to increase with incubation temperature in various oviparous reptiles, including turtles (*Chelydra serpentina*, family Trionychidae and family Emydidae) and can be negatively associated with shorter incubation periods (Du *et al*., 2010, 2011). However, these studies evaluated heart rate in a laboratory with a wide range of incubation temperatures (20.0–33.5°C; Du *et al.*, 2010, 2011), so it is possible we did not achieve similar results due to the smaller range of mean incubation temperatures (29.8–33.0°C) experienced in our naturally fluctuating *in situ* nests. Additionally, heart rate measurements have typically been established in embryos, which might not correspond to heart rates post-emergence. However, altered hydration status during incubation has been found to reduce embryonic mass, modify morphological phenotype and result in bradycardic (abnormally slow heart rate) embryos of the American alligator (*Alligator mississipiensis*; Plummer *et al*., 2003; Tate *et al*., 2012). Therefore, early-stage dehydration can result in thicker blood with higher viscosity (Wells and Baldwin, 1994), which may lead to decreased heart rate, since the heart has to pump with reduced oxygen availability to circulate thicker blood (Stone *et al*., 1968).

### Warmer nests produce slower hatchlings

The results of this study also demonstrated that righting response time is significantly and negatively associated with incubation temperatures. Similar findings have been observed in studies examining righting response on land and in other locomotor performance tests, such as crawling and swimming (Burgess *et al*., 2006; Ischer *et al*., 2009; Booth and Evans, 2011; Booth *et al*., 2012; Fisher *et al*., 2014; Sim *et al*., 2015). However, this is the first study to examine a righting response in water, the implications of which are important as hatchlings can be overturned in the near-shore waves. Hatchlings from nests laid in May had a faster righting response time, but since nesting season for loggerheads in Florida does not typically peak until the end of June/early July, it is probable that most hatchlings produced on these beaches have less than optimal righting response times or at least less rapid than early season loggerhead hatchlings. Additionally, the cooler incubation temperatures experienced in nests in April and May will likely rise with the threat of increasing nest temperatures due to climate change (Monsinjon *et al*., 2019). This could result in the majority of nests producing hatchlings with decreased locomotor abilities (Fisher *et al*., 2014; Monsinjon *et al*., 2019). It is important to note that the self-righting response test took place after blood collection, and although safe blood collection practices were followed, the potential influences of handling and blood collection on performance cannot be ruled out; however, all hatchlings were sampled in the same manner/order and these standardized protocols likely ruled out any confounding effects of blood sampling.

Following the recommendations of Booth (2017), we attempted to analyze heart rate, heart size and hemoglobin concentrations as possible underlying causes for reductions in locomotor performance. Heart rate was observed not to be significantly associated with righting response time, indicating that it might not contribute to differences in locomotor performance. However, since the righting time experienced in the water was a rapid activity, heart rate might still influence other prolonged activities that require aerobic metabolism and thus effective delivery of oxygen by the cardiovascular system, such as digging out of the nest or during the frenzy period (Booth, 2017). Too few dead-in-nest hatchlings were available during our nest excavations to determine heart size; however, hemoglobin was determined to be positively associated with locomotor performance (i.e. hatchlings with higher hemoglobin concentrations took longer to right themselves), contradicting what was expected. It was anticipated that higher hemoglobin concentrations would result in faster righting times due to higher oxygen carrying capacity in the blood. However, we attributed the observed higher hemoglobin concentrations to altered hydration status or other hemodynamic derangements. It is possible that the altered hydration status resulted in hatchlings of suboptimal quality and fitness, leading to individuals with slower self-righting response times.

## Conclusions

Climate is changing worldwide, and sea turtles, as ectotherms, are one group of organisms that are particularly vulnerable to our changing environment (Hawkes *et al*., 2009; Fuentes *et al*., 2011). This research demonstrated that higher *in situ* incubation temperatures have the potential to adversely affect hatchlings from warmer nests due to increased risk of predation from smaller body sizes, decreased physical responses and overall fitness and altered hemodynamic balance (e.g. dehydration). In the face of climate change, the fundamental information provided in this study could be vital in helping to formulate effective management plans for loggerhead sea turtles in Florida, USA. Without action, a decrease in effective population size has been projected (Hamann *et al*., 2010; Monsinjon *et al*., 2019). Increasing incubation temperatures represent just one of the multitudes of threats of climate change that will influence nesting beach habitats and the survivability of sea turtles. As these threats increase, management strategies will need to use an integrative approach of mitigation measures in order to address these multifaceted and cumulative conservation concerns and to protect not only sea turtles and their nests but also the beaches their vulnerable embryos rely upon.
